# Supplementary material for: Influence of Ovarian Status and Steroid Hormone Concentration on Day of Timed Artificial Insemination (TAI) on the Reproductive Performance of Dairy Cows Inseminated with Sexed Semen
Source: Animals (Basel). 2023 Mar 1;13(5):896. doi: 10.3390/ani13050896 (PMC10000115; doi:10.3390/ani13050896)
Supplement: Supplementary file 1 [file animals-13-00896-s001.zip › animals-2175495-supplementary.pdf]

Supplementary Materials

Table S1. Main factors influenced PF size and E_2 concentration in dairy cows inseminated with sex-sorted semen (ANOVA main effects analysis).

**Effects**	**Test**	**Value**	**Observed power (alpha=0.05)**	**P**
**Pregnancy status**	Wilks	0.579022	0.938244	0.001866
**Ovarian status**	Wilks	0.645694	0.853979	0.006536
**Estrus synchronization treatment**	Wilks	0.709251	0.732046	0.01924

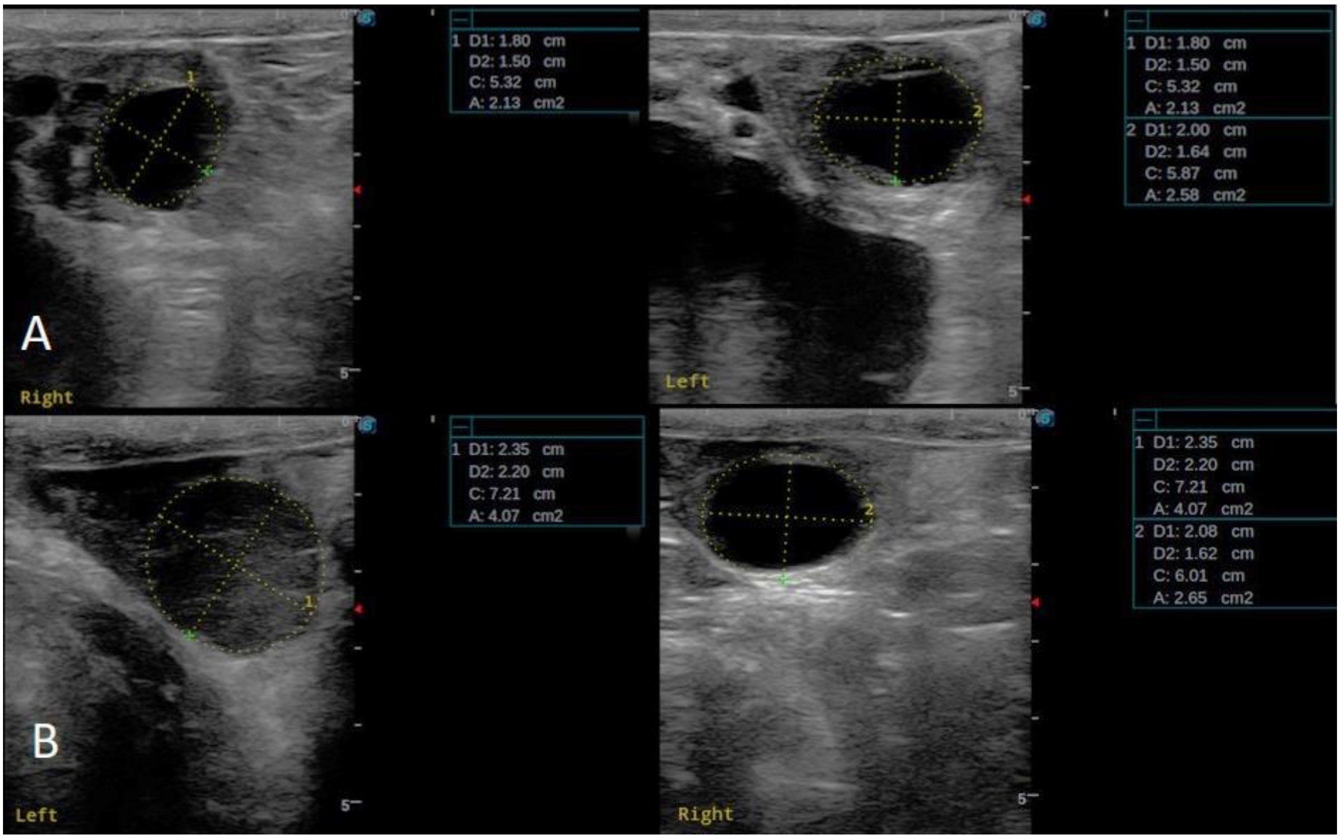

Figure S1. Ultrasound image of ovarian status in dairy cows on day of TAI (A - Preovulatory follicles in both ovaries without corpus luteum; B - Corpus luteum located in the left ovary and preovulatory follicle in the right ovary.

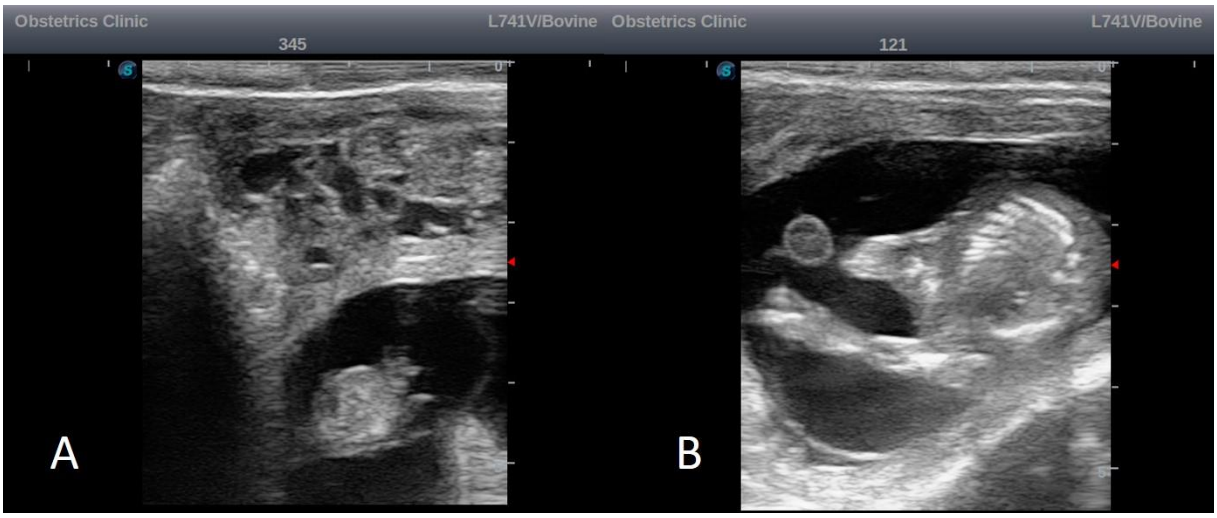

Figure S2. Ultrasound image of pregnancy in dairy cows on day 30 (A) and 60 (B) after TAI with sexed semen.
